# A Complex Case of Langer–Giedion Syndrome, Cornelia de Lange Syndrome Type 4, and Hereditary Multiple Osteochondromas with Mosaic 8q23.1–q24.12 Deletion

**DOI:** 10.3390/genes17020175

**Published:** 2026-01-31

**Authors:** Samuel David Amio Valientes, Hua Wang

**Affiliations:** 1 Division of Medical Genetics, Department of Pediatrics, Stanford Health Care, Stanford, CA 94304, USA; sdaval97@stanford.edu; 2 Division of Genetics, Department of Pediatrics, Loma Linda University School of Medicine, Loma Linda, CA 92350, USA

**Keywords:** Langer–Giedion syndrome, Cornelia de Lange syndrome, mosaic deletion, 8q23.1–q24.12, *TRPS1*, *RAD21*, *EXT1*, hereditary multiple osteochondromas, bisphosphonate, pamidronate

## Abstract

Langer–Giedion syndrome (LGS), also known as trichorhinophalangeal syndrome type II (TRPS II; OMIM #150230), is a contiguous-gene deletion disorder caused by haploinsufficiency of *TRPS1* and *EXT1*. Cornelia de Lange syndrome (CdLS) is genetically heterogeneous; heterozygous variants in *RAD21* cause the milder CdLS type 4 phenotype (OMIM #614701). Because *RAD21* lies between *TRPS1* and *EXT1*, overlapping phenotypes may arise when all three genes are deleted. We report a unique case of a 4-year-old female presenting with a blended phenotype of Langer–Giedion Syndrome (LGS) and Cornelia de Lange Syndrome (CdLS) type 4. This case is distinct from previously reported 8q deletions in three key aspects: (1) Complex Genomic Architecture: Chromosomal microarray revealed a novel complex rearrangement consisting of a 13.01 Mb mosaic interstitial deletion at 8q23.1–q24.12, flanked by two large duplications (21.5 Mb at 8q11.23–q23.1 and 25.78 Mb at 8q24.12–q24.3). (2) Rare Mosaicism: This represents only the second reported case of mosaicism affecting this contiguous gene region. Notably, the patient demonstrates a “mosaic rescue” effect, where the mosaicism appears to have mitigated the neurodevelopmental phenotype (the patient is bilingual and ambulatory) while failing to protect the skeleton. (3) First Bone-Specific Therapy: The patient suffered from severe, recurrent fractures due to a synergistic “double hit” of *TRPS1*-related osteopenia and *EXT1*-related exostoses. We report the first successful use of bisphosphonate therapy (pamidronate) in this specific mosaic profile, which resulted in a complete cessation of fractures during a 12-month follow-up. This case underscores the utility of detailed microarray analysis in complex phenotypes and suggests bisphosphonates as a viable rescue therapy for refractory syndromic osteoporosis.

## 1. Introduction

Langer–Giedion syndrome (LGS), also known as Trichorhinophalangeal syndrome type II (TRPS II), is a rare autosomal dominant contiguous-gene deletion disorder involving *TRPS1* (8q23.3) and *EXT1* (8q24.1) [1,2,3]. The phenotype combines features of TRPS I—craniofacial dysmorphism and cone-shaped epiphyses—with hereditary multiple exostoses type I due to *EXT1* haploinsufficiency and multiple osteochondromas [4,5,6]. The shortest region of deletion overlap has been localized near 8q24.11, spanning *TRPS1* and *EXT1* [1,3].

Clinically, TRPS is divided into three types. TRPS I results from TRPS1 variants and features skeletal abnormalities, sparse hair, thick eyebrows, a bulbous nasal tip, long philtrum, thin upper lip, and large ears [7,8]. TRPS II (LGS) reflects larger deletions including *TRPS1* and *EXT1* and adds multiple exostoses and, often, intellectual disability [9]. TRPS III arises from specific *TRPS1* missense variants and is associated with severe short stature and brachydactyly without exostoses [10]. LGS prevalence is ~1:100,000 [11].

Cornelia de Lange syndrome type 4 (CdLS-4; OMIM #614701) is an autosomal dominant cohesinopathy caused by heterozygous pathogenic variants or deletions involving *RAD21* [12,13]. CdLS-4 is generally milder than classic *NIPBL*-related CdLS and is characterized by synophrys, arched eyebrows, a low anterior hairline, anteverted nares, long philtrum, thin upper lip, growth delay, and variable neurodevelopmental involvement [12,13,14]. *RAD21* encodes a core component of the cohesin complex, which plays a critical role in sister chromatid cohesion, DNA repair, transcriptional regulation, and apoptosis [15].

Importantly, *TRPS1*, *RAD21*, and *EXT1* lie in close genomic proximity on chromosome 8q23.3–q24.11, creating the potential for blended phenotypes when deleted together. *TRPS1* encodes a GATA-type transcription factor that regulates chondrocyte differentiation by repressing *RUNX2* and *IHH*, thereby influencing endochondral ossification and bone mineralization [16,17]. *EXT1*, which forms a hetero-oligomeric complex with *EXT2*, is essential for heparan sulfate biosynthesis, and its haploinsufficiency leads to the formation of osteochondromas [4,5]. Loss of *RAD21* contributes primarily to craniofacial, growth, and neurodevelopmental features through disruption of cohesin-mediated chromatin organization and gene regulation [12,14,15].

Deletions spanning 8q23.1–q24.12 may encompass additional genes—such as *KCNQ3* and *CSMD3*—and thereby broaden the phenotypic spectrum to include epilepsy, central nervous system anomalies, and endocrine or metabolic manifestations [18,19,20,21]. Mosaicism further modulates clinical severity and tissue-specific expressivity, complicating genotype–phenotype correlations [22,23]. Prior reports of non-mosaic and complex rearrangements involving this region have demonstrated overlapping LGS and CdLS features [18,19,24].

Here, we describe a highly distinctive case characterized by a novel complex genomic architecture consisting of a mosaic interstitial 8q23.1–q24.12 deletion flanked by two large duplications. This report contributes to the literature in three key ways: (1) it documents a previously unreported duplication–deletion–duplication rearrangement within the LGS/CdLS spectrum; (2) it represents only the second reported case of mosaicism affecting the *TRPS1–RAD21–EXT1* contiguous gene interval; and (3) it provides the first clinical evidence supporting the use of bisphosphonate therapy, informed by prior experience in TRPS-associated osteoporosis [25], in a patient with this specific mosaic blended phenotype and recurrent fractures.

## 2. Case Description

**Figure 1:**
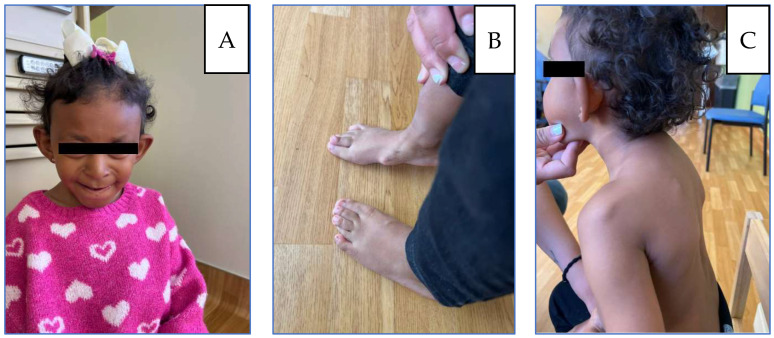
(**A**) Patient at 5 years: microcephaly, large protruding ears, broad nasal bridge, underdeveloped alae nasi, wide low-hanging columella. (**B**) Flat feet, Bilateral overlapping toes, and osteochondromas located on the internal side of the foot. (**C**) Thoracic kyphosis and winged scapulae with posterior protrusion of the superomedial left scapula and acromioclavicular region.

The patient first presented to the genetics clinic at age two (21 December 2021) for evaluation of multiple congenital anomalies and an abnormal chromosomal microarray suggestive of an unknown genetic syndrome. She was born at 39 + 3 weeks to a 30-year-old father and a 25-year-old mother. The pregnancy was notable for maternal alcohol use until 6–7 weeks of gestation, after which it was discontinued. Developmentally, she began walking independently at 16 months, and language milestones were achieved on time; she is bilingual (English/Spanish). She had a history of multiple congenital features, including microcephaly, plagiocephaly, post-axial polydactyly, overlapping toes on the left foot, and dysmorphic facial features. Her surgical history included correction of right hip dysplasia and repair of post-axial polydactyly. She had sustained a right femur fracture three months before the initial visit. On physical examination, she was noted to have large ears, a flat nasal bridge, a bulbous nasal tip, a long philtrum, and a thin upper lip; extremities were not examined (Figure 1).

A bone survey from an outside hospital revealed an acute angulated comminuted fracture of the right distal femoral metadiaphysis. There was a bony deformity involving numerous long bones with multiple sessile osteochondromas, likely secondary to underlying multiple hereditary exostoses. Postoperative changes were consistent with right proximal femoral osteotomy without evidence of hardware complications. In addition, there was an asymmetric appearance of the feet with soft-tissue prominence along the medial aspect of the right midfoot, correlating with the physical examination. No abnormalities of the axial skeleton or cone-shaped epiphyses were noted. Family history was significant for Noonan syndrome in a maternal half-cousin and polydactyly in the paternal great-grandmother. Paternal history was otherwise limited, and consanguinity was denied.

**Figure 2:**
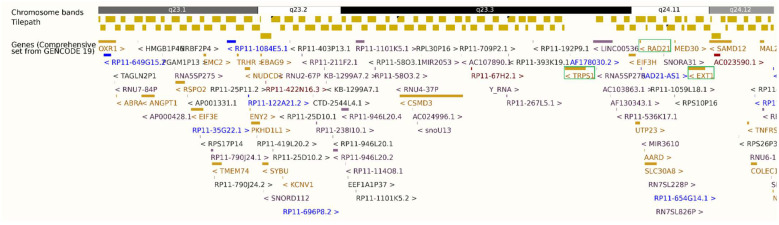
Ensembl GRCh37.p13 export showing the 8q23.1–q24.12 deletion (hg19: 8:107,431,257–120,443,762) and proximity of *TRPS1*, *RAD21*, and *EXT1* (8q23.3–q24.11), highlighted in green boxes. Gene font colors represent different biotypes: gold for protein-coding genes, blue for non-coding RNA/antisense transcripts, and red/purple for pseudogenes.

Chromosomal microarray (13 October 2021) revealed: (1) a 21.5 Mb mosaic interstitial duplication at 8q21.2–q23.1, (2) a 13.01 Mb mosaic interstitial deletion at 8q23.1–q24.12, and (3) a 25.78 Mb mosaic terminal duplication at 8q24.12–q24.3 (Figure 2 and Figure S1, Table S1). Approximately 10% of nucleated blood cells carried the duplications, while ~75%—predominantly granulocytes—harbored the interstitial deletion. The deleted interval included *OXR1*, *ANGPT1*, *RSPO2*, *TRHR*, *PKHD1L1*, *TRPS1*, *RAD21*, *SLC30A8*, *EXT1*, *SAMD12*, *TNFRSF11B*, and *COLEC10* (Table 1, Figure 2). Maternal microarray was normal. Father was not available for the test.

**Figure 3:**
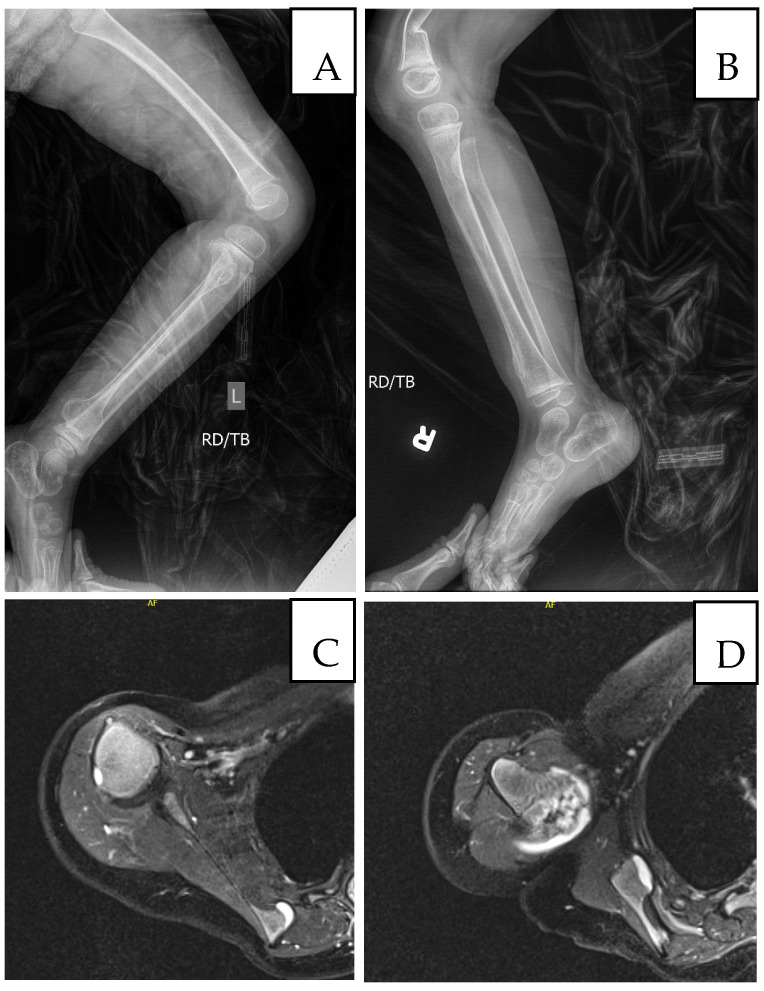
Imaging at age 5: (**A**) Left femur/tibia/fibula X-ray with sessile osteochondromas; (**B**) right tibia/fibula X-ray (comminuted distal right femur fracture shown); (**C**) MRI, medial border of left scapula; (**D**) MRI, head of left humerus and lateral left scapula.

At age three, she sustained a left wrist fracture; at age four prior to returning to the genetics clinic (24 October 2023), she sustained a left distal radial and distal right clavicular fracture. During the visit, the mother reported that she exhibited distractibility and reduced speech clarity but no other significant global developmental concerns. Overlapping toes, intoeing, and bone pain had worsened. She had a history of low muscle tone and strength for which she was receiving physical and occupational therapy. She subsequently underwent excision of a left femoral osteochondroma. Growth parameters tracked between the 10th and 50th percentiles for girls in the general population, but between the 80th and 95th percentiles relative to Cornelia de Lange syndrome norms. On physical exam, besides microcephaly, protruding ears, bulbous nose, thin upper lip as described in the previous visit, additional findings including sparse, wiry, and coarse scalp hair, synophrys with lateral thinning of eyebrows, prominent nasal bridge, short upturned nose, anteverted nares and low-hanging columella, thin upper lip, long philtrum, downturned corners of the mouth, overlapping toes, thoracic kyphosis and winged scapulae with posterior protrusion of the superomedial left scapula and acromioclavicular region, flat feet with overlapping toes and osteochondromas located on the internal side of the foot (Figure 1). MRI revealed non-expansile osteochondromas at the right humeral head and scapula; radiographs confirmed bilateral hip dysplasia and additional osteochondromas (Figure 3). A new distal right clavicle fracture was documented. A lower-limb length discrepancy was present (right femur 28 cm; left 25.5 cm).

Given her history of frequent fractures, an osteogenesis imperfecta and bone-fragility panel was performed to rule out osteogenesis imperfecta. The results confirmed a pathogenic *TNFRSF11B* deletion within the 8q-deleted interval and identified a maternal *LRP5* variant of uncertain significance (VUS) without clinical evidence of osteopetrosis. A composite genetic diagnosis of Langer–Giedion syndrome, Cornelia de Lange syndrome type 4, and hereditary multiple osteochondromas with mosaic 8q23.1–q24.12 deletion was established. Subsequent metabolic evaluation showed serum calcium, 25-hydroxy-vitamin D, osteocalcin, urine creatinine, and bone turnover markers (NTx) all within reference ranges. Dual-energy X-ray absorptiometry (DEXA; Hologic Horizon Apex 5.5.31) revealed lumbar spine (L1–L4) bone mineral density = 0.375 g/cm^2^, Z = −2.6.

## 3. Management and Follow-Up

Given her fracture history and low bone mineral density, the patient’s mother provided consent for the off-label use of pamidronate therapy. Treatment was initiated at 1 mg/kg every four months, along with vitamin D_3_ supplementation in accordance with pediatric bone-health protocols. The management plan includes annual DEXA scanning, ongoing fracture surveillance, and orthopedic follow-up for monitoring of osteochondromas. The patient has remained fracture-free for 12 months since initiation of bisphosphonate therapy.

## 4. Discussion

### 4.1. Genotype–Phenotype Correlation: The Unique Complex Architecture

Unlike classic Langer–Giedion syndrome (LGS), which typically results from simple interstitial deletions, our patient presents with a complex chromosomal rearrangement consisting of a central 13.01 Mb deletion flanked by two large duplications. This “duplication-deletion-duplication” architecture is distinct from previously reported 8q deletions and likely stems from a complex post-zygotic replicative error, given the mosaic distribution. While the phenotype is driven primarily by the haploinsufficiency of the deleted genes (*TRPS1*, *EXT1*, *RAD21*), the contribution of the flanking trisomic regions (duplications) to the atypical features cannot be ruled out, making this a genotypically unique entry in the 8q deletion spectrum.

Deletions in the 8q23.1–q24.12 region produce a complex phenotypic spectrum ranging from LGS to Cornelia de Lange syndrome type 4 (CdLS-4) [3,19]. As summarized in Table 1 and Table 2, the extent of phenotypic overlap is largely dictated by the deletion size, gene content, and specific genetic architecture. Classic LGS/TRPS II is characterized by sparse hair, distinctive facial dysmorphism, multiple exostoses, short stature, and mild-to-moderate intellectual disability. The core genotype involves contiguous deletions of *TRPS1*, *EXT1*, and *RAD21* [2,3], with the minimal critical region estimated at approximately 3.2 Mb [3]. Larger deletions generally correlate with broader clinical involvement, including more pronounced intellectual disability and congenital anomalies [2,3].

The interaction between these loci creates a blended phenotype. Overlapping features are observed when deletions encompass the critical regions for both LGS and CdLS-4—specifically *TRPS1*, *EXT1*, and *RAD21*—resulting in combined skeletal, ectodermal, and neurodevelopmental abnormalities [18,19,23]. Cases deleting *EXT1* and *RAD21* but sparing *TRPS1* may exhibit a mild LGS-like phenotype combined with CdLS-4 traits, suggesting potential disruption of *TRPS1* regulatory domains [24]. Conversely, when *RAD21* is preserved, CdLS features are typically absent even when *TRPS1* and *EXT1* are lost [28]. Furthermore, complex rearrangements, including chromothripsis, can generate atypical syndromes that complicate genotype–phenotype interpretation [23].

Finally, additional genes within the 8q23.1–q24.12 interval—such as *KCNQ3* and *CSMD3*—may contribute to neurological and central nervous system manifestations [19,21]. Atypical findings, including coccygeal agenesis, osteoporosis, and endocrine abnormalities, have been reported in larger or more complex deletions, emphasizing that both the extent and content of the chromosomal loss influence the final phenotype [18,24].

**Table 2:** Genotype–Phenotype Correlations. Comparison of case reports and studies involving deletions, including at least one gene that is responsible for our combined phenotype.

Reference	Genotype (Deleted Genes, Region, Size)	Clinical Features (LGS/TRPS II)	Clinical Features (CdLS-4)	Overlapping or Additional Features	Fracture History and Bisphosphonate Treatment
Our patient	13.01 Mb; 8q23.1–q24.12 deletion; *TRPS1*, *EXT1*, *RAD21*, 9 other genes with OMIM phenotypes	Protruding ears, bulbous nose, prominent philtrum, exostoses of ribs and scapulae, fracture risk	Synophrys, thin upper lip, polydactyly	Short stature, microcephaly, broad nasal bridge, multiple exostoses	Frequent fractures of long bones; subsequently treated with Pamidronate.
Chen et al., 2013 [19]	8q23.3–q24.22 deletion; *TRPS1*, *EXT1*, *RAD21*, *KCNQ3*; size not specified (large)	Lax skin/joints, sparse hair, facial dysmorphism, multiple exostoses, scoliosis	Intellectual disability, epilepsy, cardiovascular defects	Gastroesophageal reflux, previously misdiagnosed as Ehlers–Danlos; overlap of LGS and CdLS-4 features	No reported fracture
Favilla et al., 2022 [3]	3.2 Mb (smallest reported for classic LGS); 8q23–q24 deletion; *TRPS1*, *EXT1*, *RAD21*, ±*CSMD3*	Exostoses, facial dysmorphism, skeletal anomalies, intellectual disability (variable)	CNS anomalies (if *CSMD3* deleted)	Facial and bone anomalies most frequent; CNS anomalies possible with larger deletions	No reported fracture
Pereza et al., 2015 [29]	7.5 Mb (8q23.3–q24.13); *EXT1*, *RAD21*, 30 other genes (not *TRPS1*)	Mild LGS phenotype (skeletal/facial features, exostoses)	CdLS-4 features due to *RAD21* deletion	Suggests revision of diagnosis to CdLS-4; overlap with LGS phenotype	No reported fracture
Lei et al., 2020 [23]	Complex chromothripsis; loss of *RAD21*, *EXT1*	Bone abnormality, facial dysmorphism (atypical LGS)	CdLS-4 features (intellectual disability, facial features)	Contiguous gene syndrome with both LGS and CdLS-4	No reported fracture
Cappuccio et al., 2014 [18]	8q23.3–q24.1 deletion; *TRPS1*, *EXT1*, likely *RAD21*	Intellectual disability, short stature, microcephaly, facial dysmorphism, exostoses, osteoporosis	CNS malformations, pituitary hypoplasia	Atypical findings: coccyx agenesis, osteoporosis, hyperreninemia	Osteoporosis and increased fracture risk noted; bisphosphonate use not specified
Shanske et al., 2008 [30]	19.79 Mb (8q22.3–q24.13, mosaic); *TRPS1*, *EXT1*, likely *RAD21*, 50 genes/loci	Mild facial features, cone-shaped epiphyses, exostoses, short stature	intellectual disability (if deletion extends beyond *TRPS1*/*EXT1*)	Degree of mosaicism affects severity	No reported fracture
Pereza et al., 2012 [24]	7.5 Mb (8q23.3–q24.13); *EXT1*, *RAD21*, not *TRPS1*	LGS phenotype (facial/skeletal features, exostoses), normal height, mild developmental delay	Not classic CdLS-4, but possible overlap if *RAD21* deleted	Dyslalia, premature adrenarche; variable phenotype	No reported fracture
Lüdecke et al., 1999 [2]	Shortest region of overlap: 8q24.1; *TRPS1*, *EXT1*, *RAD21*, *EIF3S3*, *OPG*, *CXIV*	LGS features (*TRPS1*, *EXT1*), variable phenotype	CdLS-4 features (*RAD21*)	Clinical variability due to additional genes	No reported fracture
Nardmann et al., 1997 [31]	~5 Mb (TRPS I); larger in TRPS II; *TRPS1*, *EXT1*, additional genes	TRPS I: normal intelligence; TRPS II: intellectual disability if >5 Mb	Not specified	Parental origin of deletion studied	No reported fracture
Carvalho et al., 2011 [32]	8q23.1–q24.12 deletion; *TRPS1*, *EXT1*, possible others	LGS with bilateral tibial hemimelia	Not specified	Suggests additional gene(s) in region may influence limb development	No reported fracture
Schinzel et al., 2013 [28]	8q24.11–q24.13 deletion; *EXT1*, *TRPS1*	Ectodermal dysplasia, cone-shaped epiphyses, exostoses, mild intellectual impairment	Seizures, borderline cognitive delay	Joint stiffness/laxity, growth hormone/TSH deficiency, gynecomastia, vaginal atresia	Joint laxity and instability may predispose to trauma/fractures; no reported fracture
Kaur et al., 2023 [20]	Variable; *NIPBL*, *SMC1A*, *SMC3*, *HDAC8*, *RAD21*, others	Not applicable	Growth/developmental delay, limb involvement, hypertrichosis, cardiac/GI/craniofacial features	*RAD21* variants cause milder CdLS phenotype	No reported fracture
Abarca-Barriga et al., 2023 [33]	Intragenic heterozygous deletion in *RAD21* (exons 9–14)	Not applicable	Microcephaly, cleft palate, polydactyly, short stature, triangular facies, frontal bossing, bulbous nose, overfolded helix, limited pronosupination, anomalous uterus	No neurodevelopmental disorder at diagnosis; variable expressivity	No reported fracture
Maya et al., 2021 [21]	8q24.13–q24.3 deletions; variable gene content and size	High phenotypic variability; some cases with bone anomalies, some asymptomatic	Some cases with neurodevelopmental delay	No clear association between gene content and fracture risk	Fracture history not systematically reported; bisphosphonate use not described

### 4.2. Comparison of Mosaic Cases–The “Mosaic Paradox”: Dissociation of Tissue Severity

Shanske et al. (2008) [30] described the only previously reported mosaic case involving this region: a 14.5-year-old girl with a larger 19.79 Mb interstitial deletion at 8q22.3–q24.13. That deletion encompassed *TRPS1*, *EXT1*, *RAD21*, and approximately 50 other genes. Notably, mosaicism was detected in only 7% of peripheral blood lymphocytes but 97% of skin fibroblasts. Clinically, that patient exhibited mild facial features, classic skeletal manifestations (cone-shaped epiphyses, multiple exostoses, short stature), and mild intellectual disability. The authors proposed that the high level of mosaicism in fibroblasts (ectoderm/mesoderm) explained the classic skeletal phenotype, while the lower burden in other tissues may have attenuated the severity [30].

By comparison, our patient harbors a 13.01 Mb mosaic deletion at 8q23.1–q24.12, involving *TRPS1*, *EXT1*, *RAD21*, and additional genes (including *TNFRSF11B* and *COLEC10*). In contrast to the Shanske case, our patient exhibited a significantly higher burden of mosaicism in peripheral blood (~75%). Phenotypically, this correlated with a severe skeletal presentation, including multiple osteochondromas, hip dysplasia, kyphosis, winged scapulae, and the unique history of recurrent fractures. However, despite the large deletion size, which typically predicts severe intellectual disability, her neurodevelopmental profile is relatively preserved; she is bilingual with no global delay, although she exhibits distractibility and reduced speech clarity.

This comparison illustrates a ‘mosaic paradox’ or tissue-specific dissociation. Our patient’s high mosaicism in blood (mesodermal origin) correlates with her severe skeletal/hematologic fragility (‘double hit’ fractures). Conversely, her relative cognitive preservation suggests that the neuroectoderm (brain) may retain a higher proportion of normal cells than the blood, acting as a ‘mosaic rescue’ for neurodevelopment. This aligns with literature suggesting that larger deletions and higher mosaic levels in clinically relevant tissues increase the likelihood of classic TRPS II features, whereas lower levels in specific lineages can attenuate the phenotype [34]. This case underscores that in mosaic 8q syndromes, peripheral blood analysis may accurately predict skeletal severity but underestimate neurodevelopmental potential [3,18,30].

### 4.3. Skeletal Complications: The “Double Hit” Hypothesis and Therapeutic Targeting

Skeletal complications, including osteoporosis and increased fracture risk, are well recognized in Langer–Giedion syndrome (LGS/TRPS II), particularly among individuals with extensive exostoses and joint laxity [18,28]. Long-term follow-up studies have reported joint instability, trauma proneness, and, in some cases, Perthes disease or vertebral osteomas [28]. However, systematic data on fracture frequency remain limited, and most case reports do not describe bone-specific interventions.

The severity of the fracture history in our patient suggests a synergistic “double hit” mechanism compromising bone integrity. First, haploinsufficiency of *TRPS1* impairs chondrocyte differentiation and mineralization, leading to generalized osteopenia (weak material). Second, *EXT1* loss results in the formation of osteochondromas. We hypothesize that these exostoses act as biomechanical “stress risers”—focal points of structural weakness—which, when superimposed on a demineralized skeleton, precipitate fractures even with minimal trauma.

This mechanical synergy necessitates the pharmaceutical strengthening provided by bisphosphonates. Notably, Macchiaiolo et al. (2013) [25] reported a patient with TRPS and severe osteoporosis complicated by multiple spontaneous femoral fractures who showed marked improvement in bone mineral density and a complete absence of new fractures following treatment with intravenous neridronate. Consistent with this, our patient’s favorable outcome following pamidronate therapy supports the consideration of bisphosphonates as a viable adjunctive treatment for the management of refractory bone fragility associated with LGS/TRPS II and related contiguous gene syndromes.

### 4.4. Therapeutic Implications

To our knowledge, this is the first report of bisphosphonate therapy in a patient with this specific mosaic contiguous gene syndrome. The stabilization of bone density and the cessation of fractures in our patient suggest that bisphosphonates are an effective option. However, given the complexity of the condition, we propose this as a bone-specific rescue therapy for patients with refractory fractures, rather than a universal protocol. Furthermore, while the immediate clinical response (fracture cessation) has been positive, long-term follow-up is required to determine if bisphosphonate therapy can significantly improve bone mineral density (Z-scores) in this specific syndrome, or if its primary benefit lies solely in stabilizing bone turnover. In addition, assessment of clinical improvement with bisphosphonate therapy should be pursued in further cases of combined LGS/CdLS with osteoporosis to confirm our observations.

## 5. Conclusions

In conclusion, this case expands the phenotypic and genotypic spectrum of chromosome 8q disorders by documenting a novel mosaic complex rearrangement with a unique “duplication-deletion-duplication” architecture. As only the second reported instance of mosaicism in this region, this case highlights a potential “mosaic rescue” effect where tissue-specific mosaicism dissociates skeletal severity from neurodevelopmental outcomes. Most significantly, we report the first successful application of bisphosphonate therapy in a mosaic LGS/CdLS overlap. The patient’s fracture-free interval suggests that pamidronate can be an effective bone-specific intervention for the synergistic “double hit” skeletal pathology seen in this rare subset of patients. While promising, long-term follow-up is warranted to evaluate the sustained efficacy and potential effects on bone mineral density.

## Figures and Tables

**Table 1:** List of OMIM disease-associated genes in the mosaic deletion interval. The information is sourced from the UCSC Genome Browser (2025 Update) using the Table Browser tool [26,27]. OMIM genes in the deletion interval without a phenotype association were omitted from the final table.

Genes Affected	OMIM ID	Known Associations
*OXR1*	605609	Cerebellar hypoplasia/atrophy, epilepsy, and global developmental delay
*ANGPT1*	601667	Hereditary Angioedema
*RSPO2*	610575	Humerofemoral hypoplasia with radiotibial ray deficiency, Tetraamelia syndrome
*TRHR*	188545	congenital nongoitrous hypothyroidism
*PKHD1L1*	607843	Autosomal Recessive Deafness
*TRPS1*	604386	Trichorhinophalangeal syndrome type III, Trichorhinophalangeal syndrome type I
*RAD21*	606462	Cornelia de Lange syndrome type 4, Mungan syndrome
*SLC30A8*	611145	Susceptibility to Noninsulin-dependent Diabetes mellitus
*EXT1*	608177	Multiple Exostoses type 1, Chondrosarcoma
*SAMD12*	618073	Familial adult myoclonic Epilepsy
*TNFRSF11B*	602643	Juvenile-onset Paget disease of bone
*COLEC10*	607620	3MC syndrome

## Data Availability

All data supporting the findings of this study are contained within the article and its accompanying table and figures. Additional clinical details are available from the corresponding author upon reasonable request, in accordance with patient confidentiality regulations.

## Supplementary Materials

The following supporting information can be downloaded at: https://www.mdpi.com/article/10.3390/genes17020175/s1. Supplementary Table S1: List of OMIM disease-associated genes in the mosaic duplication intervals. Supplementary Figure S1: Ensembl GRCh37.p13 export showing (A) the 8q21.2–q23.1 duplication (hg19: 8:85,840,724–107,396,172) with 10% mosaic frequency and (B) the 8q24.12–q24.3 duplication (hg19: 8:120,514,645–146,295,771) with 10% mosaic frequency.
