# Supplementary figures and images for: A Complex Case of Langer–Giedion Syndrome, Cornelia de Lange Syndrome Type 4, and Hereditary Multiple Osteochondromas with Mosaic 8q23.1–q24.12 Deletion
Source: Genes (Basel). 2026 Jan 31;17(2):175. doi: 10.3390/genes17020175 (PMC12940567; doi:10.3390/genes17020175)

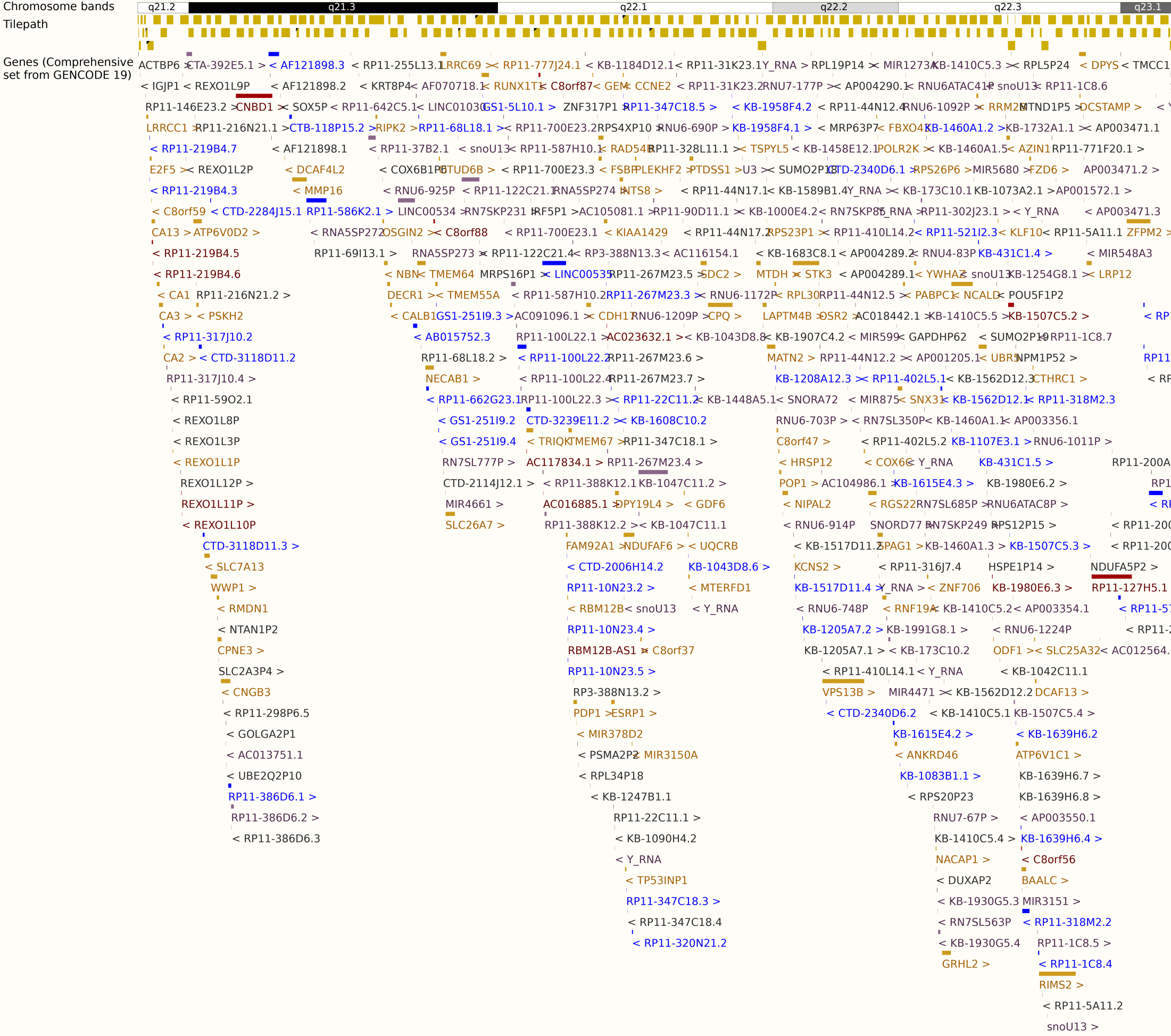

[illegible]

Supplement: Supplementary file 1 [file genes-17-00175-s001.zip › Supplementary Figure S1.pdf]
